# The NMDA Receptor Antibody Paradox: A Possible Approach to Developing Immunotherapies Targeting the NMDA Receptor

**DOI:** 10.3389/fneur.2020.00635

**Published:** 2020-07-03

**Authors:** Deborah Young

**Affiliations:** ^1^Molecular Neurotherapeutics Laboratory, Department of Pharmacology and Clinical Pharmacology, The University of Auckland, Auckland, New Zealand; ^2^Centre for Brain Research, The University of Auckland, Auckland, New Zealand

**Keywords:** GluN1, immunotherapy, NMDA receptor, neuroprotection, stroke, epilepsy

## Abstract

N-methyl-D-aspartate receptors (NMDAR) play a key role in brain development and function, including contributing to the pathogenesis of many neurological disorders. Immunization against the GluN1 subunit of the NMDAR and the production of GluN1 antibodies is associated with neuroprotective and seizure-protective effects in rodent models of stroke and epilepsy, respectively. Whilst these data suggest the potential for the development of GluN1 antibody therapy, paradoxically GluN1 autoantibodies in humans are associated with the pathogenesis of the autoimmune disease anti-NMDA receptor encephalitis. This review discusses possible reasons for the differential effects of GluN1 antibodies on NMDAR physiology that could contribute to these phenotypes.

## Introduction

Antibody-based immunotherapies form a key component of the pharmacological arsenal for treatment of cancer (1), and inflammatory diseases (2), with profound clinical success achieved for these conditions. Monoclonal antibody therapies have several desirable attributes over traditional small molecule drugs including long half-lives and high specificity for the target molecular disease driver leading to reduced off-target toxicity and a lower adverse effect profile. The pipeline of immunotherapies for central nervous system disorders is not as extensive and has largely been dominated by active or passive immunization approaches for Alzheimer's disease and Parkinson's disease that aim to modify disease progression by targeting proteins implicated in disease pathogenesis (3). Different strategies have been employed including using antibodies to neutralize the actions of putative neurotoxic protein species or to promote clearance of the offending disease protein. Clinical trials have shown some promise (4), but much work is still required to improve the therapeutic efficacy of these approaches.

The potential of antibodies to modulate the function of other molecular targets in the central nervous system (CNS) for therapeutic benefit has not been extensively investigated. In this review, I will provide an overview of our studies and those of others exploring the possibility of an immunoprotective approach for neurological diseases including stroke and epilepsy involving antibody-mediated targeting of the N-methyl-D-aspartate (NMDAR) subclass of glutamate receptor.

## The NMDA Receptor

The NMDAR plays a pivotal role in brain development, neuronal survival, and synaptic plasticity associated with learning and memory. The receptor is a hetero-tetramer composed of two obligatory GluN1 subunits of which there are eight distinct splice variants, and two variable subunits from the GluN2 (GluN2A-2D) or GluN3 (GluN3A-3B) subunit families. The combination of GluN1 with different GluN2/3 family members provides for the creation of diverse NMDAR subtypes varying in their regional distribution and functional properties. The majority of native NMDAR are triheteromeric, with GluN1/GluN2A/GluN2B receptors being the most common subtype in forebrain excitatory neurons (5).

The subunits are transmembrane-spanning and arranged to form an ion channel pore that is gated in a ligand- and voltage-dependent manner. The extracellular regions of the receptor resembling two clamshell structures with binding sites for glutamate on the GluN2 subunit and sites for glycine binding on the GluN1 subunit. The interaction between the distal amino terminal domain (ATD) of the receptor and other proteins regulate subtype-specific receptor assembly and receptor trafficking and sites for allosteric modulation of NMDAR function are also found in the ATD. The cytoplasmic C-terminus domain engages in interactions with scaffold proteins and intracellular messenger systems in the postsynaptic density.

The importance of NMDAR in the maintenance of physiological brain function is underpinned by observations that NMDAR-mediated hypofunction caused by either receptor loss, or altered distribution at synapses, is implicated in neurodevelopmental (autism spectrum disorders) (6) and neuropsychiatric disorders (schizophrenia) (7). Moreover, excessive glutamate release that leads to NMDAR overactivation contributes to neurodegeneration in acute or chronic neurodegenerative diseases including Alzheimer's disease (8, 9). The centrality of the NMDAR in the pathophysiology of a broad range of conditions makes these receptors an attractive drug target but human trials of NMDAR antagonists of different compound classes and at different sites of receptor action have been disappointing and are associated with a narrow therapeutic index and an unacceptable adverse effect profile (10). Greater insight into NMDAR function, and the discovery that synaptic and extrasynaptic NMDAR may be differentially linked to cell survival vs. cell death pathways, respectively has contributed to ongoing efforts to develop subunit-selective NMDAR antagonists. Weaker GluN2B-selective blockers that may preferentially target extrasynaptic NMDAR have a much-improved side-effect profile in humans than early generation broad spectrum antagonists (11). Other approaches to amplify the NMDAR-mediated cell survival signaling warrant investigation.

### The NMDA Receptor as an Immunotherapeutic Target

We previously described an immunotherapeutic approach for stroke and epilepsy involving targeted vaccination against the GluN1 subunit of the NMDAR (12). Rats genetically immunized to express a full-length GluN1 subunit protein developed high-titer serum GluN1 autoantibodies and were more protected in rat models of temporal lobe epilepsy and stroke. Systemic injection of the neurotoxin kainate has been used extensively to induce seizure activity and a pattern of selective neuronal cell loss in the hippocampus that recapitulates the neuropathological features observed in human temporal lobe epilepsy (13). We found that following a challenge with kainate, fewer GluN1-vaccinated rats (22 vs. 68% control-vaccinated rats) developed seizures and of the two animals that experienced 45 min of prolonged status epilepticus, only one showed evidence of neuronal cell death in the hippocampus. Moreover, in a middle cerebral artery occlusion model of ischaemic stroke, infarct lesion sizes for the GluN1-vaccinated animals were significantly smaller compared to the control-vaccinated animals following infusion of endothelin-1 (12). We did not detect any evidence of cell-mediated immune responses suggesting the protective phenotype is likely to be GluN1 antibody-mediated. Moreover, GluN1 IgG was detected at low levels in the cerebrospinal fluid (CSF) of GluN1-vaccinated rats under basal conditions prior to any insult and GluN1 antibodies are bound to antigen suggesting low-level passage across an intact blood brain barrier (BBB) (12). It has been estimated that 0.1% of systemic IgG are able to traffic through the BBB into the brain parenchyma (14). In individual animals, we found that GluN1 antibodies reacted preferentially with a few specific extracellular epitopes rather than a broad range of epitopes. To identify regions of importance, we immunized rats with recombinant GluN1 peptides that contribute to various functional domains of the NMDAR (15). Differential effects on seizure expression and injury between the different GluN1 peptide treatments were observed. These results also confirmed the protective phenotype is not a unique feature of the immunization approach used. Almost no hippocampal cell death was observed in rats immunized with a peptide consisting of amino acids 654–800 of GluN1 (GluN1[654–800]) despite extensive kainate-induced seizures sufficient in duration and intensity to induce neuronal cell death. In contrast, rats immunized with a GluN1 peptide covering amino acids 21–375 (GluN1 21–375) was associated with reduced seizure severity as assessed by a 5-point seizure rating scale following kainate challenge but hippocampal cell death was clearly evident in these rats. Expression of heat shock protein 70 (HSP70) and brain-derived neurotrophic factor (BDNF) protein were elevated by ~1.5-fold in the brains of the GluN1[654–800]-vaccinated animals that were protected against neuronal cell death compared to the control animals (naïve and Homer 1a immunized) suggesting that GluN1 antibody-mediated effects at NMDAR leads to downstream upregulation of signaling pathways linked to cell survival. These results indicate that GluN1 antibodies to specific functional domains of the NMDAR are able to induce a state of tolerance to insult akin to preconditioning whereby short-term exposure to NMDAR antagonists (16, 17) or NMDAR activation (18) can induce a state of resistance to subsequent insult.

Our studies suggest that a GluN1 immunotherapy could have broad utility for a range of neurodegenerative disorders but further mechanistic characterization is required to assess the feasibility and safety of such an approach. This is of critical importance as within the last decade, NMDAR autoantibodies targeting the GluN1 subunit have been linked to the pathogenesis of the autoimmune disease NMDAR encephalitis.

### Autoimmune Diseases Associated With NMDAR Antibodies

Anti-NMDAR encephalitis is a devastating autoimmune condition characterized by the onset of psychiatric manifestations including psychosis, rapid memory loss and seizures and the presence of high-titer CSF autoantibodies of the IgG class against the GluN1 subunit (19–21). The condition is more prevalent in women, and associated with the ectopic expression of NMDAR proteins in ovarian teratoma although there are also affected individuals who do not have detectable tumors (21, 22). The clinical features in patients and animal models resemble those caused by genetic or pharmacological attenuation of NMDAR function. Indeed, evidence from studies examining the effect of patient antibodies in cell and animal models have led to the hypothesis that the clinical syndrome is as a result of NMDAR hypofunction at a network level. Patient GluN1 autoantibodies cross-link NMDAR expressed on cultured neurons that triggers their loss at the synapse by internalization at extrasynaptic sites. Similarly, cerebroventricular infusion of patient NMDAR antibodies into rodent brain decreases NMDAR expression levels leading to impaired synaptic plasticity that is associated with memory deficits, anhedonia, depression-like behavior, and a low seizure threshold (23–26). Depleted NMDAR expression is consistent with observations in post-mortem brain from humans with anti-NMDAR encephalitis (23, 24, 27). The effects of patient antibodies are specific to NMDAR as no effect on expression of AMPA receptors or other synaptic proteins are found (27, 28).

Whilst the role of GluN1 autoantibodies in disease pathogenesis has been the key focus, more recently a mouse model of NMDAR encephalitis involving active immunization with intact native-like NMDAR GluN1/GluN2B tetramers embedded in a liposome scaffold has been described that recapitulates a broader range of features reminiscent of that found in the human disease (29). Immunized mice developed overt neurological signs include marked hyperactivity and stereotypic motor features including tight circling, seizures, and a hunched posture, or lethargy as early as 4 weeks, with nearly all animals showing abnormal behaviors by 6 weeks. This was associated with infiltration of peripheral immune cells and neuroinflammation by 6 weeks as supported by increased immunoreactivity to markers of plasma cells, CD4-positive T cells, and CD20-positive B cells, activated microglia, and astrocytes gliosis. Neuronal loss was rare. Serum autoantibodies that target epitopes on GluN1 was predominant but reactivity to GluN2 subunits as well as a peptide that lacked the amino-terminal domain of GluN1 was also observed by Western blot in the mice tested suggesting a polyclonal response by the time fulminant symptoms were present at 6 weeks after immunization. Chronic exposure of cultured hippocampal neurons to serum autoantibodies reduced NMDAR protein expression and associated NMDAR-mediated currents without an effect on synapse numbers (29). Studies of NMDAR encephalitis in humans has focused on the role of the autoantibodies, but this study suggests that mature T cells are also involved in causing a more complex disease pathogenesis leading to broader repertoire of symptoms by promoting neuroinflammation and potentiating B cell- and plasma cell–mediated antibody responses. The use of conformationally stable NMDAR holoproteins may be a critical component in initiating a more complex pattern of immunogenicity.

### The NMDAR Autoantibody Paradox

The pathogenic effects induced by patient antibodies contrast sharply to the protective benefit achieved in our studies in rodent models. Single amino acid substitutions at key residues within the extracellular regions of the GluN1 subunit can significantly affect channel permeability (30), so it is entirely plausible that site-specific targeting by GluN1 antibodies to different extracellular regions on the NMDAR could have differential effects on receptor function or distribution. Our observations showing distinct differences between effects on seizure expression and neuroprotective effects following immunization with different GluN1 peptide fragment provide support for this hypothesis (15). Using a library of peptides that span the entire 938 amino acids of the native GluN1 subunit as a screening platform, we found that GluN1 IgG antibodies from individual rats genetically vaccinated with GluN1 cDNA react most commonly with peptides that correspond to domains that form part of the extracellular vestibule of the NMDA receptor channel, including regions important for glycine binding (12). Similarly, we found neuroprotection was associated with GluN1 antibodies targeting the GluN1 [654–800] region that contributes to the S2 loop of the glycine binding domain (15). We developed a recombinant protein consisting of the extracellular pre-TM1 region that includes the amino-terminal domain (ATD) linked to the extracellular loop between TM3-4 domains of GluN1 and immunized groups of rats with this recombinant protein (recGluN1). We found that the humoral response following immunization with this protein generated GluN1 antibodies that preferentially reacted with peptides that correspond to domains important for glycine binding when we screened an IgG fraction purified from pooled rat serum against our GluN1 peptide library. Structural modeling predicts that the binding of GluN1 antibody to this target region would promote closure of the NMDAR ion channel (31).

In contrast, NMDAR patient autoantibodies recognize conformational epitopes at the GluN-ATD (28). Screening of patient autoantibodies against a series of GluN1 protein deletion mutants showed amino acid residues N368/G369 at the GluN1-ATD were crucial for the creation of reactivity of patient antibodies. Moreover, patient antibodies did not immunostain a GluN1 protein lacking the ATD, suggesting that these antibodies do not target regions important in glycine binding (28). The GluN1-ATD is a major locus for interactions between the NMDAR and various synaptic proteins that regulate the trafficking, surface distribution, and function of NMDAR (32, 33). Any biologic agent or drug compound capable of modifying these interactions could have significant effects on NMDAR signaling. Mechanistically, NMDAR encephalitis patient antibodies block the ability of Ephrin B receptors to regulate synaptic NMDAR numbers (33), leading to their depletion and a state of NMDAR hypofunction (20, 27, 34).

Conversely, neuroprotection in mouse models of stroke and experimental autoimmune encephalitis can be produced using GluN1 antibodies that target the interaction site of the serine protease tissue plasminogen activator (tPA) at the ATD (35–37). GluN1 antibodies directed against an epitope at amino acids 163–192 as well as Glunomab, a monoclonal antibody that interacts with the lysine residue at position 178, blocks the tPA-mediated potentiation of NMDAR-mediated signaling and excitotoxicity in neurons by reducing the surface dynamics and clustering of extrasynaptic NMDAR (36–39). The therapeutic beneficit engendered by these GluN1 antibodies are not restricted to actions at neuronal NMDAR, with Glunomab shown to promote the maintenance of blood brain barrier integrity via actions on NMDAR expressed on endothelial cells (36, 37). Of note, in our own work we found GluN1 antibodies that interact with the glycine site on NMDAR expressed on platelets can inhibit platelet function and thrombus formation that could also contribute to limiting stroke-induced neuronal damage (31) suggesting any therapeutic benefit could occur through additive effects at multiple cell sites. Further investigation is required to understand the full spectrum of effects on therapeutic GluN1 antibodies including the impact on NMDAR-dependent processes such as learning and memory. GluN1-ATD antibodies have been reported to impair hippocampal-dependent spatial memory in rodents (35, 39, 40) although a later study suggested that the GluN1-ATD antibodies are not associated with cognitive or behavioral deficits (36).

Altogether, these data suggest that NMDA receptor location, and function, can be differentially modulated by GluN1 antibodies in a target-dependent manner with GluN1 immunotherapeutic benefit made feasible through strategic targeting to defined sites. What are the challenges for applying such an approach, for example, as a preventative treatment against stroke-induced damage in humans?

### Challenges for a GluN1 Immunotherapy—the Role of GluN1 Autoantibodies in Health and Disease

In preclinical studies, GluN1 antibodies generated following immunization of naïve animals are presumed to be able to freely interact with their target site following passage into the brain. How the therapies would perform in humans with preexisting serum antibodies directed against the NMDAR that could directly compete for the same epitope targets (if present in sufficient quantities), is unknown. Serum GluN1 autoantibodies are found in healthy older adults and there is increased seroprevalence (>20%) in individuals affected by a wide-range of diseases including stroke, neuropsychiatric illnesses, and dementia (41–45), with a recent study suggesting GluN1 autoantibodies may be part of the normal autoimmune repertoire (46). The significance of these antibodies in contributing to functional outcomes in these conditions is an area of current investigation. Unlike NMDAR encephalitis that is primarily associated with the occurrence of IgG GluN1 antibodies, GluN1 IgA, and IgM antibodies are mainly found in non-specifically in healthy older adults and in disease conditions (44, 47). There are contradictory reports that GluN1 antibodies promote NMDAR internalization irrespective of immunoglobulin class and epitope, whereas other groups find these effects are only produced by NMDAR encephalitis-associated GluN1 IgG antibodies (44, 46, 48), suggesting further investigation into any possible pathogenic effects is required.

GluN1 autoantibodies in stroke have been associated with larger (45) as well as reduced lesion sizes after acute ischemic stroke (47). The discrepancy between these findings could depend antibody titer as well as the health of the BBB. Using apolipoprotein E4 (APOE4) carrier status as a marker for a leaky BBB, the presence of preexisting serum GluN1 autoantibodies at the time of acute ischemic stroke was associated with reduced infarct sizes in individuals with an intact BBB (APOE4 +/+), however lesion sizes appeared to be the largest in APOE4 carriers with a compromised BBB (47). We speculate these findings are in line with the neuroprotection observed in rodent stroke models with an intact BBB at the time of insult (12, 36). Whether our glycine binding site targeting GluN1 antibodies promote maintenance of BBB integrity like GluN1-ATD antibodies is unknown (37). Recent data has indicated GluN1 antibody seropositivity was not associated with any long-term functional benefit at 1 year following stroke (49) but further studies are required to examine whether therapeutic benefits might be found in specific patient subgroups such as APOE4 non-carriers.

There are many outstanding questions. Whether a GluN1 immunotherapy could counteract or override any possible pathogenic effects produced by GluN1 autoantibodies or help boost the neuroprotective capability of endogenous antibodies at multiple levels including modulating NMDAR signaling at neurons, maintaining BBB health, and function remains to be determined.

### Delivery Challenges for CNS Immunotherapeutics

Another key challenge is whether sufficient amounts of antibody as one of the key drawbacks of immunotherapies for CNS disorders is the low efficiency of delivery into the brain. The BBB strictly regulates the entry of molecules including therapeutics, immune cells, and immune mediators from the systemic circulation into and out of the brain. Osmotic or chemical disruption of BBB integrity can facilitate delivery of therapeutics into the brain but the lack of specificity for the therapeutic biologic agent is problematic. Alternative methodologies have exploited the properties of endogenous BBB receptor-mediated transporters responsible for the passage of endogenous large molecules such as insulin, transferrin, insulin-like growth factor, and leptin into the brain. These circulating proteins bind to their cognate receptors on the luminal surface of the endothelial cells lining the BBB. Upon binding, the receptor–ligand complex is internalized into the endothelial cell by receptor-mediated endocytosis where the ligand molecule is transported across the abluminal membrane of the endothelial cell into the brain. Molecular Trojan horses that are engineered to carry peptides or proteins ligands that target receptor mediated transport systems (e.g., receptor-binding sequences of insulin) or monoclonal antibodies that specifically target transferrin and insulin receptors have been shown to be effective in facilitating delivery of various therapeutic proteins into the brain (50, 51). Progress in antibody engineering has led to the generation of different antibody configurations including the artificial bispecific antibody that combine two antigen-recognizing components into a single construct. Bispecific antibodies could also act as scaffolds to deliver therapeutic antibodies into the brain by incorporating one arm with specificity against a BBB receptor-mediated transport receptor that facilitates passage across the BBB and the therapeutic arm that produces the pharmacological effect (52). Use of these technologies coupled with site-specific targeting of the GluN1 could be explored in future studies if required.

## Author Contributions

DY wrote the paper and conceived this work.

## Conflict of Interest

The author declares that the research was conducted in the absence of any commercial or financial relationships that could be construed as a potential conflict of interest.
